# 

**Published:** 1900-07

**Authors:** 


### CONTENTS.

ORIGINAL COMMUNICATIONS. PAGE

Hypnotic Suggestion in the Practice of Dentistry. By WALTER H. NEALL, D.D.S., Philadelphia . . . 437

The Etiology of Pyorrhœa Alveolaris discovered so stated. By DR. B. F. ARRINGTON, Goldsboro, N. C. . . . 451

Dental Education. By DR. EUGENE S. TALBOT, Chicago, Ill. . . . 458

A New Method of inviting Sleep without Drugs. By DR. J. B. LEARNED, Northampton, Mass. . . . 462

A Response to the Toast "Dentistry." By DR. C. C. BARKER, Meriden, Conn. . 466

ABSTRACTS AND TRANSLATIONS.

Micro-Organisms in Dental Caries. By KENNETH W. GOADBY, L.D.S. (Eng.) . 471

REPORTS OF SOCIETY MEETINGS.

Academy of Stomatology . . . 484

American Academy of Dental Science . . . 493

EDITORIAL.

Is Dentistry drifting into Medicine? . . . 495

BIBLIOGRAPHY . . . 499

DOMESTIC CORRESPONDENCE.

The Value of Local Anæsthetics . . . 500

OBITUARY.

Theodore Menges, B.S., A.M., D.D.S. . . . 501

Dr. George H. Cushing . . . 503

Resolutions of Respect to Dr. George H. Cushing, M.D., D.D.S. . . . 505

Dr. Nathaniel Ware Hawes . . . 506

CURRENT NEWS.

International Dental Congress . . . 507

Missouri State Dental Association . . . 507

New York State Dental Society . . . 508

Iowa State Dental Society . . . 508

Removal . . . 508

